# Prediction of Sleep Stages Via Deep Learning Using Smartphone Audio Recordings in Home Environments: Model Development and Validation

**DOI:** 10.2196/46216

**Published:** 2023-06-01

**Authors:** Hai Hong Tran, Jung Kyung Hong, Hyeryung Jang, Jinhwan Jung, Jongmok Kim, Joonki Hong, Minji Lee, Jeong-Whun Kim, Clete A Kushida, Dongheon Lee, Daewoo Kim, In-Young Yoon

**Affiliations:** 1 Asleep Inc. Seoul Republic of Korea; 2 Department of Psychiatry Seoul National University College of Medicine Seoul Republic of Korea; 3 Department of Artificial Intelligence Dongguk University Seoul Republic of Korea; 4 Department of Psychiatry Seoul National University Bundang Hospital Gyeonggi-do Republic of Korea; 5 Department of Otorhinolaryngology Seoul National University Bundang Hospital Gyeonggi-do Republic of Korea; 6 Department of Otorhinolaryngology Seoul National University College of Medicine Seoul Republic of Korea; 7 Department of Psychiatry and Behavioral Sciences Stanford University Medical Center Redwood City, CA United States

**Keywords:** respiratory sounds, sleep stages, deep learning, smartphone, home environment

## Abstract

**Background:**

The growing public interest and awareness regarding the significance of sleep is driving the demand for sleep monitoring at home. In addition to various commercially available wearable and nearable devices, sound-based sleep staging via deep learning is emerging as a decent alternative for their convenience and potential accuracy. However, sound-based sleep staging has only been studied using in-laboratory sound data. In real-world sleep environments (homes), there is abundant background noise, in contrast to quiet, controlled environments such as laboratories. The use of sound-based sleep staging at homes has not been investigated while it is essential for practical use on a daily basis. Challenges are the lack of and the expected huge expense of acquiring a sufficient size of home data annotated with sleep stages to train a large-scale neural network.

**Objective:**

This study aims to develop and validate a deep learning method to perform sound-based sleep staging using audio recordings achieved from various uncontrolled home environments.

**Methods:**

To overcome the limitation of lacking home data with known sleep stages, we adopted advanced training techniques and combined home data with hospital data. The training of the model consisted of 3 components: (1) the original supervised learning using 812 pairs of hospital polysomnography (PSG) and audio recordings, and the 2 newly adopted components; (2) transfer learning from hospital to home sounds by adding 829 smartphone audio recordings at home; and (3) consistency training using augmented hospital sound data. Augmented data were created by adding 8255 home noise data to hospital audio recordings. Besides, an independent test set was built by collecting 45 pairs of overnight PSG and smartphone audio recording at homes to examine the performance of the trained model.

**Results:**

The accuracy of the model was 76.2% (63.4% for wake, 64.9% for rapid-eye movement [REM], and 83.6% for non-REM) for our test set. The macro F1-score and mean per-class sensitivity were 0.714 and 0.706, respectively. The performance was robust across demographic groups such as age, gender, BMI, or sleep apnea severity (accuracy 73.4%-79.4%). In the ablation study, we evaluated the contribution of each component. While the supervised learning alone achieved accuracy of 69.2% on home sound data, adding consistency training to the supervised learning helped increase the accuracy to a larger degree (+4.3%) than adding transfer learning (+0.1%). The best performance was shown when both transfer learning and consistency training were adopted (+7.0%).

**Conclusions:**

This study shows that sound-based sleep staging is feasible for home use. By adopting 2 advanced techniques (transfer learning and consistency training) the deep learning model robustly predicts sleep stages using sounds recorded at various uncontrolled home environments, without using any special equipment but smartphones only.

## Introduction

Growing knowledge that sleep plays a vital role in maintaining well-being and good health, both physical and mental, increases public interest and awareness regarding the importance of sleep to health. Therefore, the demand for knowing and taking care of one’s own sleep increases, so does the demand for sleep monitoring [1,2]. The gold-standard test for monitoring and quantifying sleep is polysomnography (PSG), which typically requires 1 night of sleep at a sleep center with various biosignals recorded, such as the electroencephalogram (EEG; brain wave activity), electrooculogram (EOG; eye movement activity), electromyogram (EMG; muscle activity), electrocardiogram (ECG; heartbeat activity), and respiratory signals. After the overnight recording, the sleep data are reviewed by human experts to score sleep stages, arousals (ie, brief awakening), and respiratory and movement events. While PSG remains the most accurate diagnostic tool for sleep, it is too expensive and inconvenient to be used for a general population on a daily basis. In addition, a standard PSG taken in a laboratory environment may not reflect one’s habitual sleep at home [3-6]. An easy and convenient method is thus required to enable home-based daily sleep monitoring for the general population [1,2].

Various commercial sleep trackers are available (ie, wearable or nearable devices), mostly using accelerometer for activity and movements, ECG or photoplethysmogram for heart rate variability, piezoelectricity or radar for respiratory movements, or EEG for brain activity [7-13]. However, because of their inconvenience and high cost, people do not vigorously use these devices. Recently, sound-based sleep staging has emerged as a new alternative, relying on recognizing sound patterns of respiratory and body movements [14-20]. The advantage of using sounds is that sleep can be measured remotely without contact [17-20]. Among the various studies performed in this regard, a deep learning model (SoundSleepNet) predicted sleep stages using smartphone audio recordings with good accuracy [20], showing the potential of sound-based sleep staging using smartphones.

However, sound-based sleep staging models have been developed and tested only in laboratory environments such as in a hospital [14-20]. Unfortunately, feasible sleep sounds are mostly recorded during PSG in hospitals, while PSG requires a controlled environment (ie, a quiet and soundproof room where the examinee stays alone). It also remains questionable as to whether a sound-based sleep staging model can work well in home environments, as diverse and dynamic background noise is present in home environments (eg, home appliances, pets, roommates, and outdoor noise such as traffic noise). Therefore, it is more difficult to train a model to predict sleep stages using home sounds, which are full of uncontrolled noise, compared with using hospital sounds. Consequently, a specific training is needed to derive a model to work at home.

An obstacle in this regard is that deep learning models require thousands of ground truth (ie, PSG for sleep measure) to be trained; however, large amounts of home PSG data are not yet available. Thus, introduction of advanced techniques may help bypass the step of collecting large-sized home PSG data. One technique that may be useful is transfer learning. It allows a model trained by hospital sounds to learn to predict sleep stages for home sounds [21-24]. Another useful technique is consistency training, using which a model can be trained with hospital sounds augmented by adding home noise. It makes a model learn to predict sleep stages regardless of the presence of home noise [25,26].

Meanwhile, validation of sleep trackers at home is important [1,2]. Most sleep trackers have only been validated in laboratory environments [7-10,14-20] because it requires a lot of effort to prospectively collect home PSG data [1,2]. However, the performance should be addressed specifically in “home environments” to really serve as daily home sleep trackers [1,2].

In this study, we propose a deep learning model adopting advanced training techniques for sound-based sleep staging at home, an uncontrolled environment full of noise. The performance of the proposed model was examined by level 2 PSGs conducted at home.

## Methods

### Sleep Sound Data Sets

#### Overview of the Data Sets

This study used 3 different data sets: a hospital PSG data set (level 1 PSG and audio recording for 812 nights) and a home smartphone data set (smartphone audio recordings without PSG for 829 nights) for training, and a home PSG data set (level 2 PSG and matched smartphone audio recordings for 45 nights) as the test data set.

#### Hospital PSG Data Set

This is a clinical data set from the sleep center of Seoul National University Bundang Hospital (SNUBH) collected between 2019 and 2020, which includes PSG and matched audio data [20]. As the data set was retrospectively collected from PSGs previously conducted, additional informed consents were not available. All data were anonymized.

#### Home Smartphone Data Set

Adult volunteers were recruited and screened through an internet survey and audio recordings were collected between June and November 2022. Informed consent was obtained from each participant by an electronic form. Audio recording at night was self-conducted by each participant using his or her own smartphone at home following predefined instructions. Various models of smartphone were used, ranging from Android (OS version later than 8.0; Google LLC/Alphabet Inc) to iOS devices (OS version later than 15; Apple Inc). The participants were asked to place the phone 0.5-1 m from their head. Using phone models owned by the participants for data collection simulates the real scenario, while recordings from various phones help the model to adapt to different microphone settings (see Multimedia Appendix 1 for additional information regarding how the participants were selected for the study).

#### Home PSG Data Set

Adult volunteers were recruited at the sleep center of the SNUBH and home PSG tests were performed together with audio recordings between June and November 2022. Written informed consents were obtained from each participant. A portable PSG setup was made by sleep technicians at the center and an iPhone 11 was provided for audio recording. Participants were asked to sleep at home during PSG, with the provided smartphone placed on a side table or mattress, with a 0.5-1.0-m distance from their head.

The demographics of the participants in each data set are presented in Table 1. Additional details of the 3 data sets are described in Multimedia Appendix 1.

**Table 1:** Demographics of the 3 data sets.

Demographics		Hospital PSG^a^ (training) data set (n=812)	Home smartphone (training) data set (n=829)	Home PSG (testing) data set (n=45)
Age^b^ (year), mean (SD)		52.7 (13.6)	36.2 (9.7)	44.7 (15.8)
Male, n (%)		562 (69.2)	330 (39.8)	19 (42.2)
BMI^b^ (kg/m^2^), mean (SD)		25.9 (4.1)	23.2 (4.3)	24.0 (3.9)
**AHI^b,c^, mean (SD)**		23.3 (23.0)	—^d^	11.8 (16.4)
	AHI<5, n (%)	193 (23.8)	—	22 (48.9)
	5≤AHI<15, n (%)	182 (22.4)	—	11 (24.4)
	15≤AHI<30, n (%)	207 (25.5)	—	7 (15.6)
	30≤AHI, n (%)	230 (28.3)	—	5 (11.1)
Rapid-eye movement sleep behavior disorder, n (%)		72 (8.9)	—	—
Restless legs syndrome, n (%)		26 (3.2)	—	—
Insomnia, n (%)		220 (27.1)	—	—

^a^PSG: polysomnography.

^b^Continuous variable.

^c^AHI: apnea-hypopnea index.

^d^Not available.

### Polysomnography

For PSG, standard sensors and channels were used (eg, 6-channel EEG, 2-channel EOG, chin EMG, ECG, 2-leg EMGs, respiratory effort, airflow, and oxygen saturation). Level 1 PSG was performed at the hospital under monitoring by sleep technologists. For level 2 PSG, after experienced sleep technologists from SNUBH hooked up participants with recording electrodes and equipment for each test, the participants went home to conduct their test at home. The main difference between level 1 and level 2 PSG is the presence of technologists and real-time monitoring during the recording [27]. After the PSG recording, sleep technologists reviewed each study and manually annotated the study for sleep stages, followed by confirmation by a sleep specialist, in accordance with the American Academy of Sleep Medicine scoring manual [28]. Each 30-second epoch of PSG was scored as 1 of 5 sleep stages, namely, wake, rapid-eye movement (REM), non-REM (NREM) stage 1 (N1), 2 (N2), and 3 (N3).

### Data Preprocessing

All audio data were cut into 30-second epochs, preprocessed by adaptive noise reduction and Mel spectrogram conversion, and matched with the corresponding PSG labels to train and verify the model [20]. In addition, pitch shifting was applied as a simple data augmentation technique. Ground truth labels were only available for the 2 data sets for which PSGs were conducted concurrently, namely, the hospital PSG data set for training and the home PSG data set for test.

### Deep Neural Network Architecture

#### Training Overview

To fairly demonstrate the effects of the training techniques proposed in this paper, we adopted the SoundSleepNet model and its trained network parameters that performed well in hospital environments [20]. The network processed 40 input Mel spectrograms of sound data, each representing one 30-second sleep epoch, and output sleep-stage predictions of the 20 middle epochs (40 to 20). By adopting a well-trained model, performance difference is guaranteed to arise from only the additional training techniques, not from the network architecture.

#### Training Components

**Figure 1:**
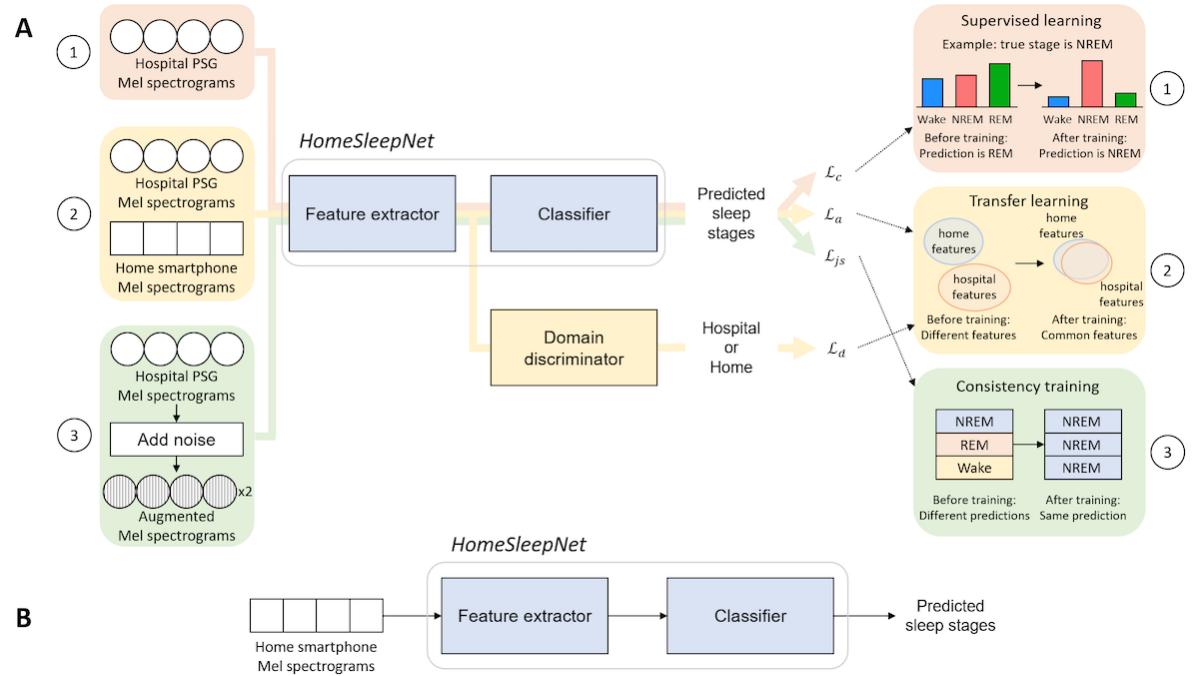
Training and inference of HomeSleepNet. (A) Training phase with 3 training components. On the left side, the data input for each training component was marked; on the right side, the impact of each component to the training is visually explained. Three training components are (1) supervised learning that trained the feature extractor and classifier to correctly predict sleep stages with Mel spectrogram inputs in hospital environments; (2) transfer learning that transferred sleep staging knowledge from hospital to home environments using a domain discriminator; (3) consistency training that helped the model make robust predictions despite the home noise presence. (B) HomeSleepNet in the inference phase after training is completed. All training blocks were removed, and only the feature extractor and classifier remained for the classification task. *L_c_*: cross-entropy loss; *L_a_*: auxiliary loss; *L_js_*: Jensen-Shannon consistency loss; *L_d_*: binary cross-entropy loss; NREM: nonrapid-eye movement; PSG: polysomnography; REM: rapid-eye movement.

The proposed model, dubbed HomeSleepNet, was trained by 3 training components (Figure 1A).

The first component was supervised learning [29,30], where the large-sized hospital PSG data set was used to train the HomeSleepNet model to make correct predictions of sleep stages from the input Mel spectrograms in hospital environments.

The second component was transfer learning [21-24], where Mel spectrograms from both hospital and home were used. Using a domain discriminator, the feature extractor was trained to transfer the sleep staging knowledge from the hospital domain to the home domain.

The third component was consistency training [25,26], for which 2 augmented hospital sound inputs were needed. Consistency training helps the HomeSleepNet model to perform sleep staging reliably in the presence of home noise.

The 3 training components were executed concurrently to preserve the effects of each component. The details of each training component are described in the following sections.

#### Supervised Learning for Sleep Staging in Hospital Environments

The purpose of the supervised learning for HomeSleepNet is to train the network with preprocessed Mel spectrograms and matched sleep-stage ground truths from the hospital PSG data set so that the network can predict sleep stages using the input Mel spectrogram data in hospital environments.

The supervised learning component was used to train 2 subnetworks: a feature extractor and a classifier (Figure 1A). The feature extractor uses Mel spectrograms of hospital sound data as input and extracts temporal and frequency features related to respiratory and sleep activity patterns. The classifier receives the features and predicts sleep stages of each Mel spectrogram. As a result, we train both the feature extractor and the classifier by minimizing the cross-entropy loss that measures the difference between the sleep-stage ground truth and the network predictions.

#### Transfer Learning via Unsupervised Domain Adaptation

Transfer learning for HomeSleepNet was executed by unsupervised domain adaptation (UDA) [21]. The goal of UDA is to make a model originally trained with a source domain (hospital environments) perform similarly for a target domain (home environments). One popular direction of UDA is to extract common features (ie, *domain-invariant features*) between data from the source domain and the target domain so that the model can perform well regardless of the domain of the input data [22-24].

**Figure 2:**
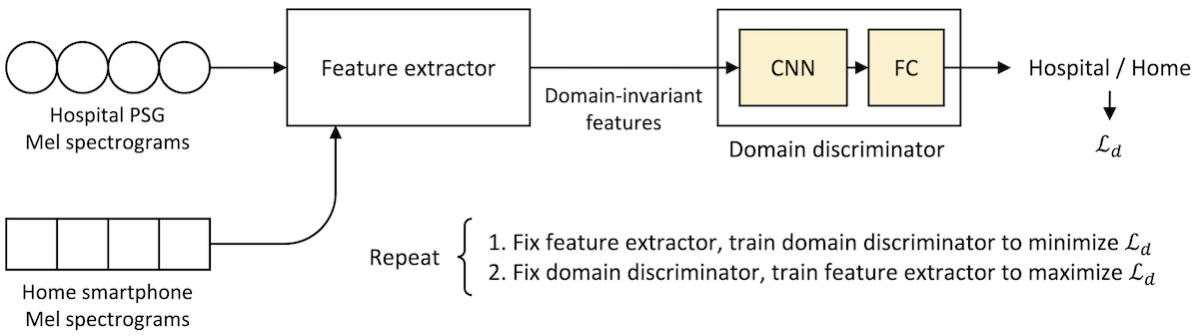
Training procedure of transfer learning (unsupervised domain adaptation). Mel spectrograms from hospital and home domains are used as training data. The feature extractor will extract only domain-invariant features when the domain discriminator is not able to classify the input domains. *L_d_* is the binary cross-entropy loss function representing the difference between the domain prediction and the domain ground truth. CNN: convolutional neural network; FC: fully-connected layer; PSG: polysomnography.

Following Ganin et al [22], we added a domain discriminator comprising simple convolutional layers followed by several fully connected layers (Figure 2). The feature extractor generates features from input Mel spectrograms, and the domain discriminator predicts the original domain of the features (hospital domain or home domain). Hospital PSG sound data and home smartphone sound data were used as inputs from the 2 domains. The training itself, however, does not require sleep-stage labels but only the domain ground truth of each input data. A binary cross-entropy loss, which indicates the difference between the domain prediction and the domain ground truth, was used to train both subnetworks. We used an adversarial training algorithm [22,24], in which the domain discriminator is trained to be more accurate (i.e., minimize the loss), while the feature extractor is trained to make the domain discriminator less accurate (i.e., maximize the loss) by extracting features that confuse the domain discriminator. In the end, the domain discriminator can no longer recognize the input domains from the extracted features, which means the extracted features are “domain invariant.” Therefore, the well-trained classifier (Figure 1) can correctly predict the sleep stages regardless of the original domain.

In addition to the adversarial training, an auxiliary loss, which consists of conditional entropy and virtual adversarial training [24], was applied (Figure 1A) to reserve the classifying performance after domain adaptation.

#### Consistency Training Using Augmented Data

In this study, we applied *consistency training* [25] to train the model to output consistent predictions on hospital data, regardless of the presence of home noise. *Data augmentation* was used for this purpose by artificially adding home noise to the original hospital data to simulate sounds recorded at home. The feature extractor and classifier were trained through consistency training to predict the same sleep stage for the augmented data as they do for the original data.

**Figure 3:**
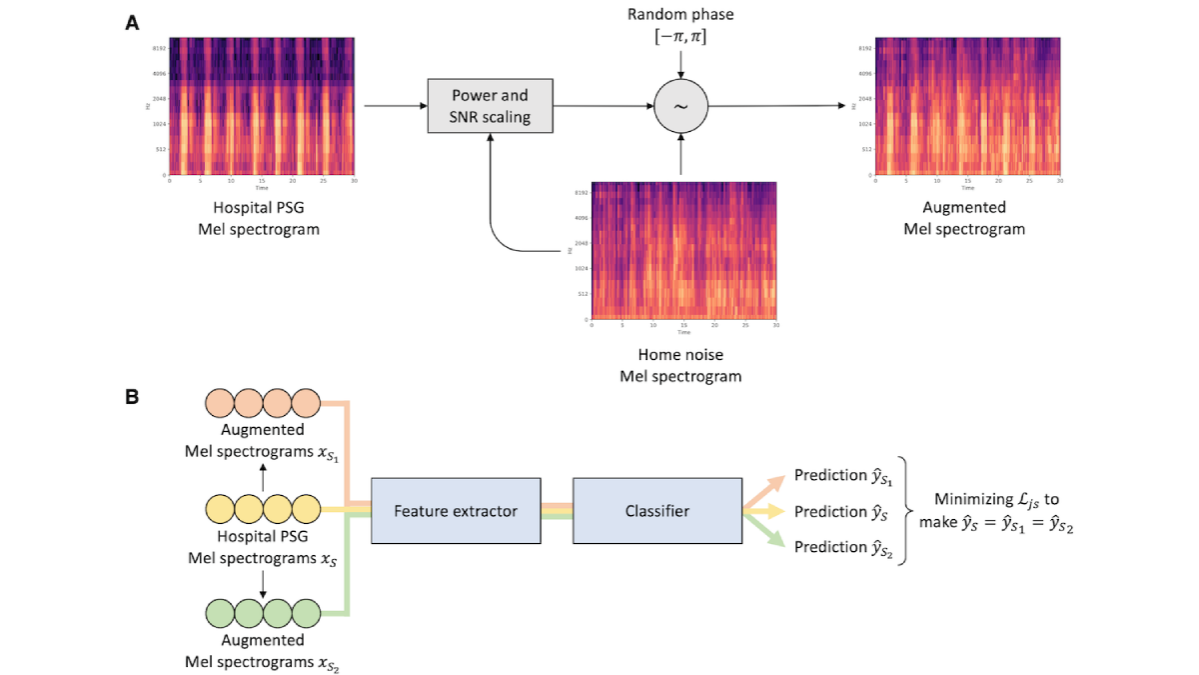
Framework of consistency training. (A) The process of creating an augmented Mel spectrogram from hospital data and home noise data. The augmented data retain breathing patterns in the clean hospital data and noise features in the home noise data. (B) The consistency training procedure. Consistency loss *L_js_* is minimized to make identical predictions on hospital data and augmented data. PSG: polysomnography; SNR: signal-to-noise ratio.

In detail, to create the augmented data, home noise audio was converted into a Mel spectrogram and added into the Mel spectrogram of hospital data with randomly generated phases and signal-to-noise ratio value ranging from –10 dB to 10 dB (Figure 3A). Noise audios were downloaded from Freesound (Music Technology Group) [31], an open database of sounds that can be used for scientific research. We used audio tags about home environments (such as home appliances, room noise, air conditioner, fan) to filter out the unrelated audio files and form a home noise data set. In total, 8255 sound clips, preprocessed identically to the sleep sound audio, were used for this research. More information on how the noise clips were filtered and downloaded is presented in Multimedia Appendix 1.

For consistency training (Figure 3B), we created 2 augmented noisy samples from the original hospital data *x_S_* by applying the noise adding process with 2 different types of home noise data. The hospital data *x_S_* and the 2 augmented samples were then fed into the feature extractor and classifier to obtain 3 corresponding predictions. We then adapted Jensen-Shannon divergence loss [26] as the consistency loss that measures the difference between the 3 predictions. By minimizing this consistency loss, the resulting model is able to generate robust and consistent predictions, even in the presence of home noise.

#### Training Settings

Pretrained network parameters from SoundSleepNet [20] were used to initialize the parameters of HomeSleepNet. The training used the Adam optimizer with a fixed learning rate of 0.0002 within 20 training epochs. The aggregated training algorithm using the 3 components is presented in Multimedia Appendix 1. In the inference phase after training, only feature extractor and classifier subnetworks remained for the sleep staging task (Figure 1B).

### Evaluation Methods

We evaluated HomeSleepNet in 4 different ways using the home PSG data set.

First, the main outcome was the sleep staging performance for the 3-stage classification (wake, REM, and NREM) with evaluation metrics of accuracy, Cohen κ, macro *F*_1_-score, and mean per-class sensitivity. Accuracy shows the overall quality of the model prediction; Cohen κ evaluates the interrater reliability between HomeSleepNet predictions and PSG sleep stages; macro *F*_1_-score evaluates the model while considering the data imbalance; mean per-class sensitivity evaluates the model predictions for each sleep stage. For all 4 metrics, the higher the score, the better the performance. Performance for the 4-stage (wake, light sleep, deep sleep, and REM) and 2-stage (wake and sleep) classifications was also reported. In 4 stages, N1 and N2 were classified as light sleep and N3 was defined as deep sleep. The principal component analysis (PCA) plots [32] were presented to show clusters in the feature space of the model. Using the output of the last hidden layer in HomeSleepNet, PCA was used to extract the most representative features of each input data in a 2D format. These extracted 2D features are then illustrated on a 2D coordinate plane. If there appear sleep-stage clusters in the plane, it means that the predictions from HomeSleepNet are reliable.

Second, multiple sleep metrics were compared between predictions of HomeSleepNet and manual annotations of PSGs. The presented sleep metrics were total sleep time, sleep onset latency, sleep efficiency, wake after sleep onset, REM latency, and portions of each sleep stage, which were all calculated per night. Total sleep time is the total time asleep, calculated by adding all 30-second epochs annotated or predicted as sleep (ie, N1, N2, N3, and REM). Sleep onset latency is the length of time between lights off and the first epoch scored as sleep. Sleep efficiency is calculated as total sleep time divided by the total time spent in the bed (in our case, the recording time). Wake after sleep onset is the total wake time between the first sleep and the last sleep epoch of the night. REM latency is the length of time between the first sleep epoch and the first REM sleep epoch. Portions of each sleep stage were calculated by the sum of each stage divided by the recording time per night. The agreement between the 2 measurements was presented by the Bland-Altman plots.

Third, to investigate performance according to demographic characteristics, we divided the test data set into groups regarding age, gender, BMI, and apnea-hypopnea index (AHI). Performance of HomeSleepNet was evaluated on each group, respectively.

Lastly, an ablation study was conducted to show the contribution of each training component in HomeSleepNet. Specifically, from the original SoundSleepNet model, we trained 2 additional variant models: one with added transfer learning only and another with added consistency training only. As a result, our final model HomeSleepNet was compared against its 3 variants: (1) SoundSleepNet, (2) SoundSleepNet with transfer learning, and (3) SoundSleepNet with consistency training. SoundSleepNet was only derived from supervised learning (the first training component) using the hospital PSG data set, without any additional techniques for training or input of home sound data [20].

### Ethical Considerations

The use of the 3 data sets (hospital PSG data set, home smartphone data set, and home PSG data set) was approved by the Institutional Review Board of Seoul National University Bundang Hospital (SNUBH; approval number B-2205-755-308). All participants signed the written consents before the data recording was performed. All the recorded data were anonymized for privacy and confidentiality protection of the participants.

## Results

### Sleep Staging Performance

**Figure 4:**
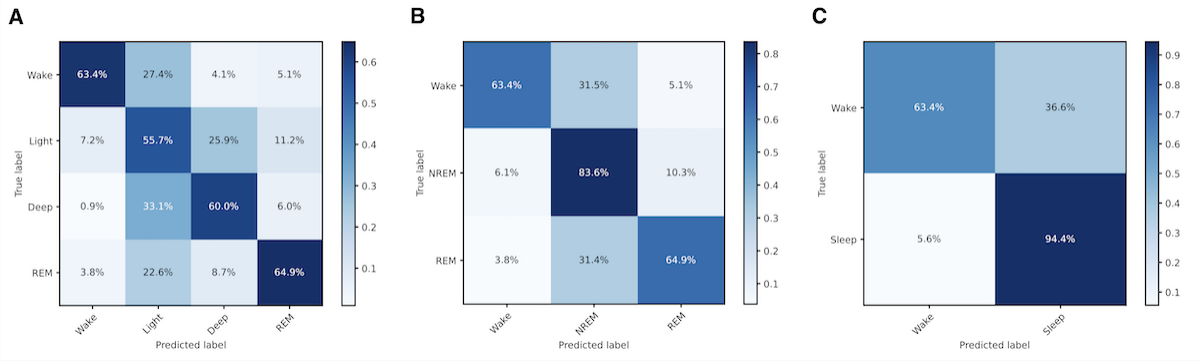
Confusion matrices showing performance of HomeSleepNet on the home PSG data set: (A) 4-stage classification, (B) 3-stage classification, and (C) 2-stage classification. Light: N1+N2; Deep: N3; NREM: N1+N2+N3. NREM: nonrapid-eye movement; REM: rapid-eye movement; PSG: polysomnography.

HomeSleepNet showed a good performance for the 3-stage classification with an overall accuracy of 76.2%. Specifically, it correctly predicted 63.4% of wake, 83.6% of NREM sleep, and 64.9% of REM sleep (Figure 4). Other metrics also showed a reasonable performance for both macro *F*_1_-score (0.714) and mean per-class sensitivity (0.706). Only Cohen κ was not as high, with a value of 0.557 (Table 2). For the 2-stage classification, all 4 metrics showed an even better performance. Accuracy of sleep-wake prediction was high, up to 88.5%. Both macro *F*_1_-score and mean per-class sensitivity were around 0.8 and Cohen κ increased to 0.610. For the 4-stage classification, the performance was not as good, with an accuracy of 59.4%.

**Figure 5:**
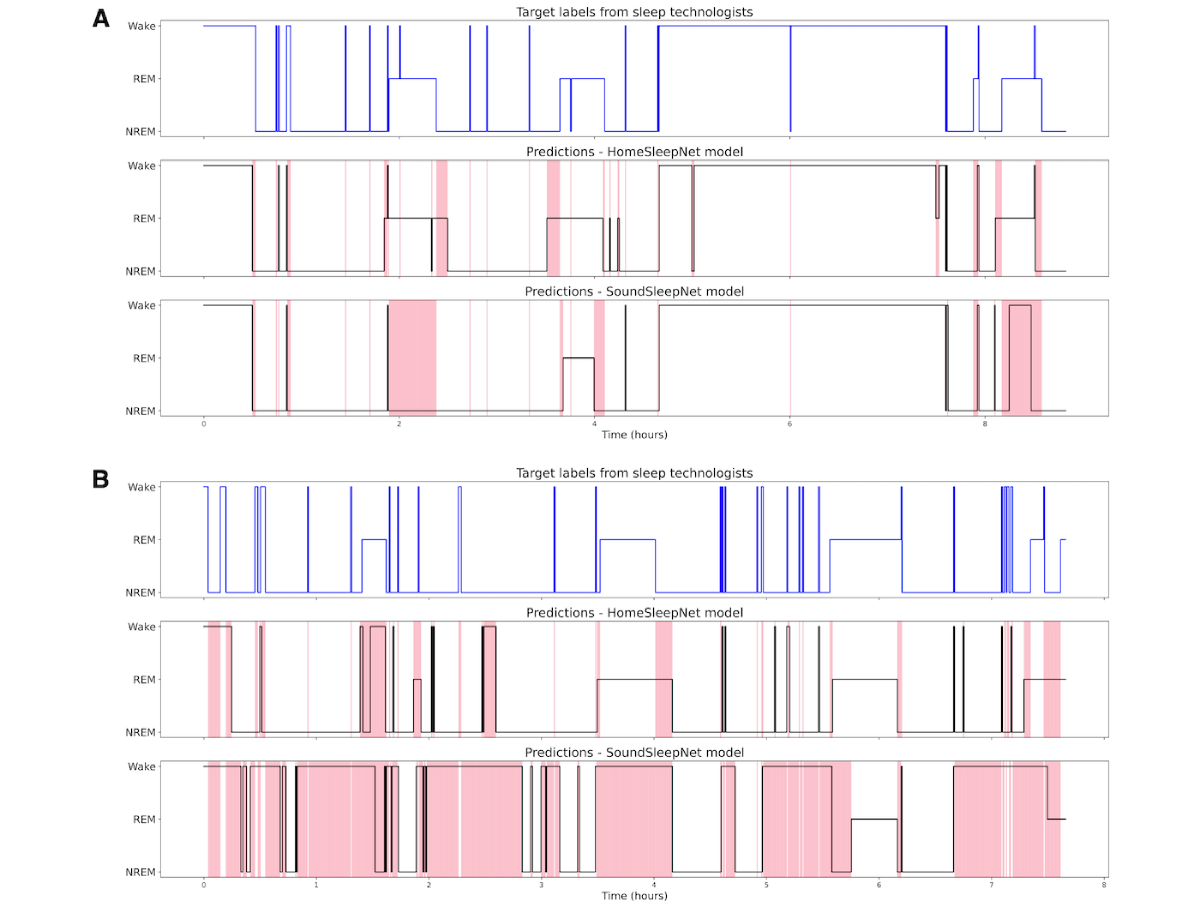
Comparison of whole-night sleep stage predictions among manual annotations of PSG (top), predictions of HomeSleepNet (middle), and predictions of SoundSleepNet (bottom) for 2 participants: (A) Male, 44 years old, BMI 24.1 kg/m^2^, AHI 47.5; (B) Female, 65 years old, BMI 23.6 kg/m^2^, AHI 1.8. The highlighted red regions indicate different predictions compared with the sleep stages from PSG. NREM: nonrapid-eye movement; PSG: polysomnography; REM: rapid-eye movement.

Figure 5 shows the whole-night sleep-stage predictions from the baseline model SoundSleepNet and our proposed HomeSleepNet for 2 participants. The first participant was a 44-year-old male with BMI of 24.1 kg/m^2^ and AHI of 47.5, and the second participant was a 65-year-old female with BMI of 23.6 kg/m^2^ and AHI of 1.8. According to the analysis on different demographic groups (discussed later), sound-based sleep staging for the first participant is expected to be easier than that for the second participant. Indeed, SoundSleepNet performed reasonably well for the first participant, although it misclassified 2 REM blocks in the middle and in the end of the sleep. However, for the second participant, SoundSleepNet did not perform well, predicting most epochs as wake which were wrong. By contrast, HomeSleepNet was able to successfully predict most sleep stages and captured the sleep transitions for both participants.

**Figure 6:**
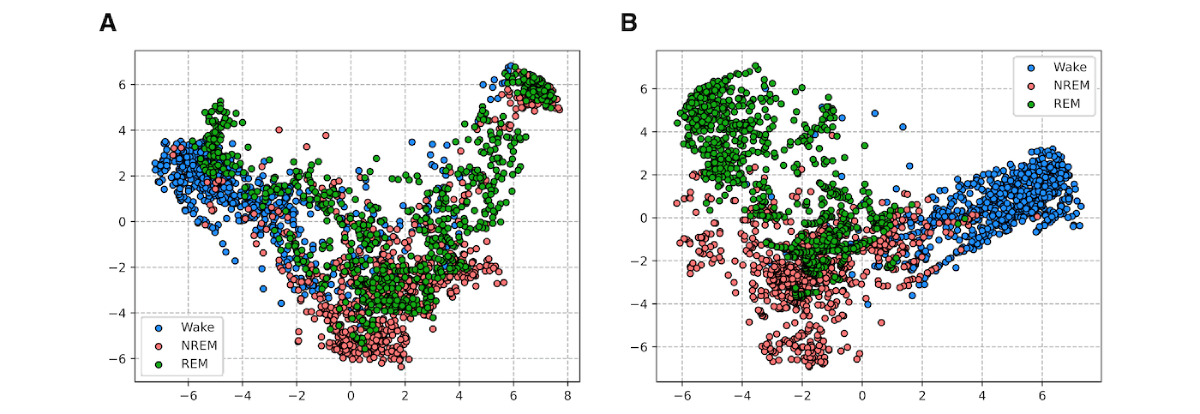
Feature space visualization using principal component analysis with 2 different models: (A) the baseline SoundSleepNet and (B) the proposed HomeSleepNet. NREM: nonrapid-eye movement; REM: rapid-eye movement.

In Figure 6, we present PCA plots [32] from the last hidden layer outputs of our proposed HomeSleepNet and the baseline SoundSleepNet models. We randomly selected 800 sleep epochs from each class for visualization (2400 sleep epochs in total), due to limited computing resources. The feature space is better organized with more clearly divided clusters in HomeSleepNet compared with SoundSleepNet. In the feature space of SoundSleepNet, data points from each class were widely scattered, especially those from the REM stage. This finding also supported the superior sleep staging ability of HomeSleepNet over SoundSleepNet using sounds recorded from home environments.

**Table 2:** Sleep staging performance of HomeSleepNet on the Home PSG^a^ data set.

Classification type	Cohen κ	Macro *F*_1_-score	Mean per-class sensitivity	Accuracy, %
4 Stage^b^	0.416	0.582	0.610	59.4
3 Stage^c^	0.557	0.714	0.706	76.2
2 Stage^d^	0.610	0.805	0.789	88.5

^a^PSG: polysomnography.

^b^Wake, light (N1+N2), deep (N3), and rapid-eye movement sleep.

^c^Wake, rapid-eye movement, and nonrapid-eye movement (N1+N2+N3) sleep.

^d^Wake and sleep (N1+N2+N3+rapid-eye movement).

### Sleep Metrics Comparison

**Figure 7:**
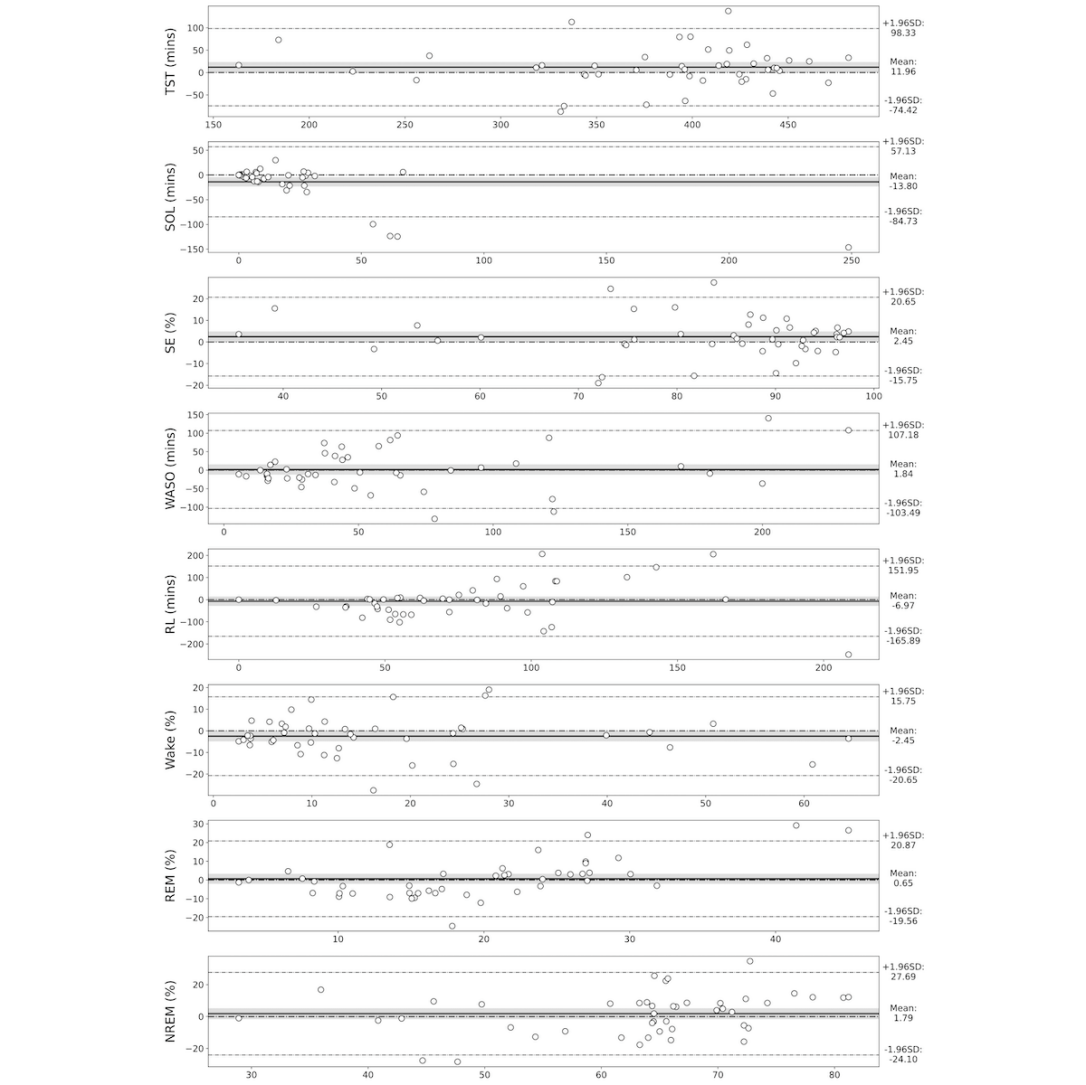
Bland-Altman plots of common sleep metrics: Total sleep time (TST), sleep onset latency (SOL), sleep efficiency (SE), wake after sleep onset (WASO), REM latency (RL), and each sleep stage portion in the 3-class setting. The x-axis represents the mean value of the 2 methods and the y-axis represents the difference values. The solid line indicates mean difference and the dash-single dotted line is the line of equality (y=0). The dashed lines indicate the 95% limit of agreement, and the shaded regions are the 95% CI of the mean difference. NREM: nonrapid-eye movement; PSG: polysomnography; REM: rapid-eye movement.

The sleep metrics calculated using the 3-stage predictions from HomeSleepNet were compared with those derived from manual annotations of PSGs (Table 3). For most sleep metrics, the mean predicted values were similar to the mean values from PSG, and the differences were relatively small. Bland-Altman plots also showed consistent agreement between the sleep metrics derived from HomeSleepNet and PSG (Figure 7). The line of equality in all graphs is located within the range of a 95% CI of the mean difference or close to the border, which suggests that there was no significant systematic difference between the 2 methods. Although HomeSleepNet presented lower averaged sleep onset latency compared with PSG, the gap was attributed to incorrect predictions for a few outliers with unusually long sleep onset latencies (eg, 5 hours). When excluding the outliers, the mean predicted sleep onset latency was similar to that of PSG. For more information, please refer to Multimedia Appendix 1.

**Table 3:** Comparison of sleep metrics between HomeSleepNet and a portable PSG^a^ device.

Sleep metrics	Portable PSG, mean (SD); 95 percentile confidence interval	HomeSleepNet, mean (SD); 95 percentile confidence interval	Difference, mean (SD); 95 percentile confidence interval
Total sleep time (minutes)	375.0 (75.0); 353.1 to 396.9	387.0 (74.9); 365.1 to 408.9	12.0 (44.1); –0.9 to 24.8
Sleep onset latency (minutes)	26.4 (53.9); 10.6 to 42.1	12.6 (27.9); 4.4 to 20.7	–13.8 (36.2); –24.4 to –3.2
Sleep efficiency (%)	81.1 (16.3); 76.3 to 85.8	83.5 (15.6); 79.0 to 88.1	2.4 (9.3); –0.3 to 5.2
Wake after sleep onset (minutes)	63.3 (56.9); 46.7 to 79.9	65.1 (67.3); 45.5 to 84.8	1.8 (53.7); –13.9 to 17.5
REM^b^ latency (minutes)	80.1 (52.7); 64.7 to 95.5	73.1 (61.6); 55.1 to 91.2	–7.0 (81.1); –30.7 to 16.7
REM (%)	19.1 (7.3); 17.0 to 21.3	19.8 (12.6); 16.1 to 23.5	0.7 (10.3); –2.4 to 3.7
NREM^c^ (%)	61.9 (11.6); 58.6 to 65.3	63.7 (14.9); 59.4 to 68.1	1.8 (13.2); –2.1 to 5.7
Wake (%)	18.9 (16.3); 14.2 to 23.7	16.5 (15.6); 11.9 to 21.0	–2.4 (9.3); –5.2 to 0.3

^a^PSG: polysomnography.

^b^REM: rapid-eye movement.

^b^NREM: nonrapid-eye movement.

### Performance on Different Demographic Groups

Among the 3 age groups, performance tended to slightly increase as age reduced (Multimedia Appendix 1). The performance of HomeSleepNet was better for men than for women. The performance was similar between the high and low BMI groups. Regarding AHI, the performance was better in people with moderate-to-severe sleep apnea (AHI≥15) than in people with no or mild sleep apnea (AHI<15). Overall, HomeSleepNet showed a robust performance in all groups, with all accuracies higher than 73%. More detailed results can be found in Table S2 in Multimedia Appendix 1.

### Ablation Study

The comparison of the sleep staging performance between HomeSleepNet and its 3 variants is presented in Table 4. As expected, the original SoundSleepNet model showed the worst performance in all evaluation metrics. Although adding only transfer learning to SoundSleepNet did not significantly improve the accuracy, transfer learning resulted in balancing the predictions between the classes, as the mean per-class sensitivity increased from 0.65 to almost 0.68. By contrast, adding consistency training to SoundSleepNet enhanced accuracy by 4.3%; besides, there were slight improvements in other metrics as well: 0.01 in mean per-class sensitivity, 0.038 in macro *F*_1_-score, and 0.05 in Cohen κ. HomeSleepNet, using both transfer learning and consistency training, achieved the best performance with an increased accuracy of 7% from SoundSleepNet as well as considerable improvements in other metrics: around 0.1 in Cohen κ, 0.08 in macro *F*_1_-score, and 0.056 in mean per-class sensitivity.

**Table 4:** Ablation study comparing HomeSleepNet with its multiple variants.^a^

Model variants	Cohen κ	Macro *F*_1_-score	Mean per-class sensitivity	Accuracy, %
SoundSleepNet	0.454	0.632	0.650	69.2
SoundSleepNet + transfer learning only	0.477	0.648	0.679	69.3
SoundSleepNet + consistency training only	0.501	0.670	0.660	73.5
HomeSleepNet	0.557	0.714	0.706	76.2

^a^All results were based on the 3-stage classification.

## Discussion

### Principal Findings

Our finding shows that sound-based sleep staging can perform well not only in the hospital but also in individuals’ home environments. Our proposed deep learning model, HomeSleepNet, was designed specifically for home sleep monitoring by adopting transfer learning and consistency training. We collected numerous home sound data and selected a large variety of home noise data from an open database, and we utilized them together with a labeled hospital sound data set for transfer learning and consistency training, respectively. The significance of HomeSleepNet is that it enables sound-based sleep staging with a good performance in home environments where a lot of background noise exists.

### Comparison With Prior Work

To the best of our knowledge, this is the first study to tackle the sound-based sleep staging problem in home environments. Early sound-based sleep staging studies were limited by the need for professional recording equipment [17,18] or short recording distances [19]. SoundSleepNet was a breakthrough in sound-based sleep staging, using sounds recorded from a distance of 1 m with only smartphone microphones, a more practical approach that still yielded good performance [20]. Although SoundSleepNet demonstrated the potential of using smartphone audio recordings for sleep staging, its real-world performance for home use remains unknown. The model was trained and tested on sounds recorded in a hospital environment and its ability to accurately classify sounds in a real-world, nonclinical setting is yet to be fully evaluated.

Therefore, this study aimed to develop a specifically designed model for home use. HomeSleepNet achieved an overall accuracy of over 75% in differentiating between wake, REM, and non-REM sleep using home sound data. This level of accuracy is similar to previous methods that used hospital sound data, which typically had lower levels of background noise [17,19,20]. For example, SoundSleepNet showed an accuracy of 79.8%, macro *F*_1_-score of 0.749, and a mean per-class sensitivity of 0.757 for the 3-stage classification using hospital data [20]. However, as can be seen in the ablation study, the same model failed to work well on home-based sounds, showing an accuracy of 69.2%, macro *F*_1_-score of 0.632, and a mean per-class sensitivity of 0.650 for the 3-stage classification, showing a significant decrease in performance.

Even for the gold-standard test, PSG, the interrater agreement of manual scoring between technologists is approximately 82%-83% for 5 sleep stages [33,34]. Regarding other methods, the mean per-class sensitivity for 4 sleep stages was 0.480-0.632 among the commercial sleep trackers and 0.655 for SoundSleepNet [20]. The mean per-class sensitivity of 0.610 should thus be considered acceptable, especially when using home sounds that are full of uncontrolled noise.

### Difference Between Hospital and Home Environments

In the real world, home-based sound data from users will be recorded by their own smartphones. A huge variety of input from home-based sounds is expected, reflecting the diversity of smartphone models, home environments, and background noise. By contrast, the data obtained in hospitals were collected under controlled conditions (noise isolation and designated devices for audio recording). The decreased performance of SoundSleepNet on home data confirms that sounds recorded from hospitals and home should be considered as different data domains. This reinforces the theory that a good performance in a controlled environment may not warrant practical use in the real world [3-6]. Although the performance of HomeSleepNet on home data might not be as reliable as its performance on hospital data, considering the challenges of using home sound data for sleep staging (ie, more noisy data and lack of ground truth), the robust performance of HomeSleepNet using home-based sound data is of great importance.

### Transfer Learning and Consistency Training

It is important to note that HomeSleepNet was trained without home PSGs. Instead, 2 specific techniques, transfer learning and consistency training, were added to solve the problem of the lack of ground truth for home data. When comparing transfer learning and consistency training, with regard to improvement of performance on home data, adding consistency training to SoundSleepNet showed a greater improvement than adding transfer learning. We have two hypothesis for this phenomenon. First, we allowed participants to use their own smartphones to record home-based sounds to better reflect real-world data. In this study, we broadly grouped all home-based sound data using different devices into 1 domain for these to be distinguishable from hospital data. However, the home smartphone data set itself is actually heterogenous; technically, each type of smartphone used for audio recording can be considered an independent domain because of its own configuration. Grouping heterogeneous data into 1 target domain might impair the domain adaptation training. Second, adapting the model from controlled data (collected by 1 designated microphone in the hospital) to uncontrolled home data (collected through various types of smartphones) might have reduced the performance of the transfer learning. However, adding both transfer learning and consistency training showed a better performance than adding only consistency training, implying that transfer learning also had positive effects on performance. Therefore, it can be concluded that both transfer learning and consistency training contribute to the enhanced performance of sleep staging in HomeSleepNet, with each method employing unique mechanisms to assisting the network training.

In general, transfer learning and consistency training can be applied to any deep learning model for better performance across various target domains. When either sleep sound data or background noise data from a target domain are sufficiently available, the proposed training methods can be used to benefit a sound-based sleep staging model. Examples of the target domains are sounds recorded in other environments or sounds recorded by other devices (eg, other types of smartphones, smart speakers, or smart televisions).

### Limitations

There are several limitations to our research. First, the performance of the proposed model was based on data that were collected with the condition that people slept alone in the room. The presence of multiple people or accompanying pets may cause overlapping or interruption of the sleep sounds and reduce the performance for sleep staging. Second, the majority of home smartphone data were collected from young healthy adults. Third, the test set might not be big enough to test performance under all diverse cases. Fourth, the HomeSleepNet model has difficulty differentiating light and deep sleep, a limitation shared by sound-based sleep staging methods [14,19,20].

### Conclusions

To the best of our knowledge, this is the first sound-based sleep staging study to utilize sounds recorded in individual home environments. The performance was validated by comparing with PSGs recorded concurrently at home. By adopting the 2 techniques for training — one that transfers learning from a hospital data to home audios, and another that ensures consistency in the presence of home noise — the proposed model is able to accurately predict sleep stages using home sounds. Our proposed model expands the use of sound-based sleep staging from in-laboratory sounds to home sounds full of uncontrolled noise. Daily sleep monitoring using the simple audio recording function of smartphones is feasible. An easy and convenient noncontact sleep tracker may encourage individuals to track their own sleep, which may further modify their awareness of and behaviors for sleep.

## Appendix

Multimedia Appendix 1 Additional information on the 3 sleep data sets used in this study, the HomeSleepNet training algorithm, and further discussion of results.

## Abbreviations

AHI: apnea-hypopnea index
NREM: nonrapid-eye movement
PSG: polysomnography
REM: rapid-eye movement
UDA: unsupervised domain adaptation

## Data Availability

The data used in this study are not publicly available. Because of privacy and security concerns, the audio data are not easily redistributed to researchers other than those engaged in the institutional review board–approved research collaborations with the named medical centers.
