# Supplementary material for: Prediction of Sleep Stages Via Deep Learning Using Smartphone Audio Recordings in Home Environments: Model Development and Validation
Source: J Med Internet Res. 2023 Jun 1;25:e46216. doi: 10.2196/46216 (PMC10273036; doi:10.2196/46216)
Supplement: Multimedia Appendix 1 [file jmir_v25i1e46216_app1.pdf]

Prediction of Sleep Stages Via Deep Learning Using Smartphone Audio Recordings in Home Environments: Model Development and Validation

Multimedia Appendix 1

Methods

Participants

Among the three sleep datasets, the hospital PSG dataset is the same as previously reported in the SoundSleepNet study [20]. PSG and matched audio data were recorded from clinical patients at the sleep center of Seoul National University Bundang Hospital (SNUBH). The PSG was conducted using the EmblaTM N7000 device (Embla, Reykjavik, Iceland), and the matched audios were recorded by microphone chips (SUPR-102, ShenZhen YIANDA Electronics Co. Ltd., Shenzhen, China) installed on the ceiling, 1.7 meters above the subject's head (**Table S1**).

Table S1. Summary of three sleep datasets and their usage in this research.

	Data domain	Audio recording device	PSG	Availability of ground truth	Number of nights	Usage in experiments
**Hospital PSG dataset**	Hospital	SUPR-102[a]	Level 1 PSG[d]	Yes	812	Train
**Home smartphone dataset**	Home	Smartphone (random various models)[b]	No PSG	No	829	Train
**Home PSG dataset**	Home	Smartphone (defined models)[c]	Level 2 PSG[e]	Yes	45	Test

[a] SUPR-102 (ShenZhen YIANDA Electronics Co. Ltd., Shenzhen, China)
[b] Volunteers used their own smart phones, ranging from Android (OS version later than 8.0) to iOS devices (OS version later than 15)
[c] iPhone 11 (Apple Inc., California, United States)
[d] EmblaTM N7000 device (Embla, Reykjavik, Iceland)
[e] Embletta MPR/ST+ proxy (Natus Medical Inc., Middleton, WI, USA)

For the other two home datasets, home smartphone dataset and the home PSG dataset, we prospectively enrolled participants and collected data between June and November 2022. First, the home smartphone dataset, volunteers were recruited and screened through an internet survey. Those who passed the screening were asked to download the mobile application specifically designed for audio recording. Instructions were provided, such as placing the phones 0.5–1.0 meter from the

subject's head, connecting the phones to chargers, and activating the recording button before sleep. Various models of smart phone were used, ranging from Android (OS version later than 8.0) to iOS devices (OS version later than 15).

For the home PSG dataset, volunteers were recruited by the sleep center of the SNUBH and written informed consents were obtained from each participant. An Embletta MPR/ST+ proxy (Natus Medical Inc., Middleton, WI, USA) was used for home PSG with standard electrodes and sensors. An iPhone 11 was provided for audio recording. Participants were asked to place the smartphone on a side table or on the mattress, with a 0.5–1.0 meter distance from their head.

The inclusion criterion for all three datasets was age 20 years or older. The two home datasets required an additional criterion that subjects needed to sleep in the bedroom, i.e., without a partner or pet, during the recording. For the large-sampled home smartphone dataset, stratification was done according to age in a ratio of 1:1:2 to the 20s, the 30s, the 40s and older, respectively.

Exclusion criteria for all three datasets were as following: (1) patients with major physical illness or psychiatric disorders; (2) patients with a history of head trauma, neurological disorders, cerebrovascular diseases, or a brain tumor; (3) incomplete audio data. The cases of incomplete audio data were: (1) a recording error which was suggested by the portion of zero values exceeding 15% of one-night audio; (2) insufficient information for the temporal synchronization of audio and PSG. For the two datasets including PSG, subjects who failed PSG or whose total sleep time from PSG were less than 240 minutes were excluded.

Forming the noise dataset

The noise clips were downloaded from Freesound using their provided Python API code given at https://github.com/MTG/freesound-python. We chose keywords and sound tags (home, room noise, fan, etc.) that are highly likely to be recorded from residential environments. The noise clips must have a user rating above 4.0 to be selected for training. In the end, we were able to form a noise dataset with 8,255 noise clips to be used for consistency training.

HomeSleepNet Aggregated Training Algorithm

Combining all three components described in the above sections gives a complete training algorithm for HomeSleepNet (**Algorithm 1**).

Algorithm 1. HomeSleepNet training algorithm

The following two steps are sequentially repeated until convergence.

Step 1: Train the classifier. Sample M hospital data $\boldsymbol{x_S}$ with M corresponding sleep stages $\boldsymbol{y_S}$, M smartphone data $\boldsymbol{x_T}$, and 2xM noise samples to update $\boldsymbol{\theta_G}$ and $\boldsymbol{\theta_H}$:

$$\min_{\theta_G, \theta_H} \mathcal{L}_c(\theta_G, \theta_H; \mathcal{X}_S, \mathcal{Y}_S) - \lambda_d \mathcal{L}_d(\theta_G; \mathcal{X}_S, \mathcal{X}_T) + \lambda_a \mathcal{L}_a(\theta_G, \theta_H; \mathcal{X}_S, \mathcal{X}_T) + \lambda_{js} \mathcal{L}_{js}(\theta_G, \theta_H; \mathcal{X}_S, \mathcal{X}_N)$$

Step 2: Train the domain discriminator. Sample M hospital data $\boldsymbol{x_S}$ and M smartphone data $\boldsymbol{x_T}$ to update $\boldsymbol{\theta_D}$:

$$\min_{\theta_D} \mathcal{L}_d(\theta_D; \mathcal{X}_S, \mathcal{X}_T)$$

We use the notation $\mathcal{L}_x(\theta_y; D_z)$ to mean that the loss $\mathcal{L}_x$ updates the parameters θ_y by using the data from D_z. θ_G represents the feature extractor parameters, θ_D means the domain discriminator parameters, and θ_H denotes parameters of the feature classifier. X_S represents the hospital audio data and Y_S denotes the corresponding sleep stage labels. Similarly, X_T represents the home smartphone dataset, and X_N is the home noise dataset. Please note that we used SoundSleepNet as the pretrained network to train HomeSleepNet.

Results

The Seeming Underestimation of HomeSleepNet in Sleep Onset Latency

The mean sleep onset latency (SOL) of Portable PSG is 26.4, while the value for HomeSleepNet predictions is 12.6, which indicates an underestimation of HomeSleepNet. The reason for the seeming underestimation of HomeSleepNet in SOL is because of several special data from the Home PSG dataset. For example, there exists this one data that has in total almost 8 hours of sleep, but the sleep stage in the first 5 hours was Wake, which results in a SOL of 5 hours. The HomeSleepNet model, though performing well overall, predicted several Light sleep stages at around 3 hours, which results in a SOL of around 3 hours, 2 hours less than the truth value from the PSG data (**Figure S1**). We tried removing the special data that has long Wake in the beginning HomeSleepNet failed to predict (6 special subjects in total) and recalculated the statistics. The new results without the special data became reasonable, as mean SOL for Portable PSG is 9.2 and mean SOL for HomeSleepNet predictions is 11.0, a difference of only 1.8.

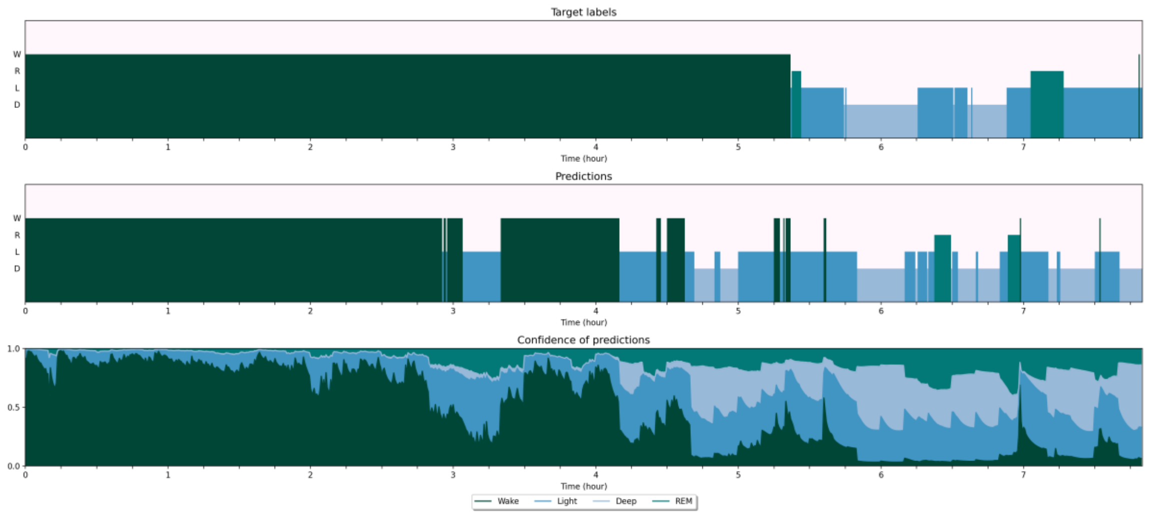

Figure S1. Illustration of special data with 5 hours of Wake from the beginning of the sleep test.

Performance on Different Demographic Groups

Table S2 and **Figures S2** below showed performance of HomeSleepNet on different demographic groups.

Table S2. Performance of HomeSleepNet according to patient characteristics.

Feature	Group	Number of patients	Number of epochs	Cohen's kappa	Macro F1	Accuracy
**Age**	19-39	23	20,760	0.594	0.740	78.0%
	40-59	11	10,580	0.542	0.702	75.3%
	60-79	11	10,480	0.497	0.673	73.4%
**Gender**	Male	19	17,220	0.594	0.728	79.4%
	Female	26	24,600	0.526	0.687	73.9%
**BMI**	< 25	28	26,520	0.576	0.724	76.6%
	≥ 25	17	15,300	0.517	0.687	75.3%
**AHI**	< 15	33	30,840	0.547	0.707	75.5%
	≥ 15	12	10,980	0.585	0.735	78.0%

BMI, body mass index; AHI, apnea-hypopnea index.

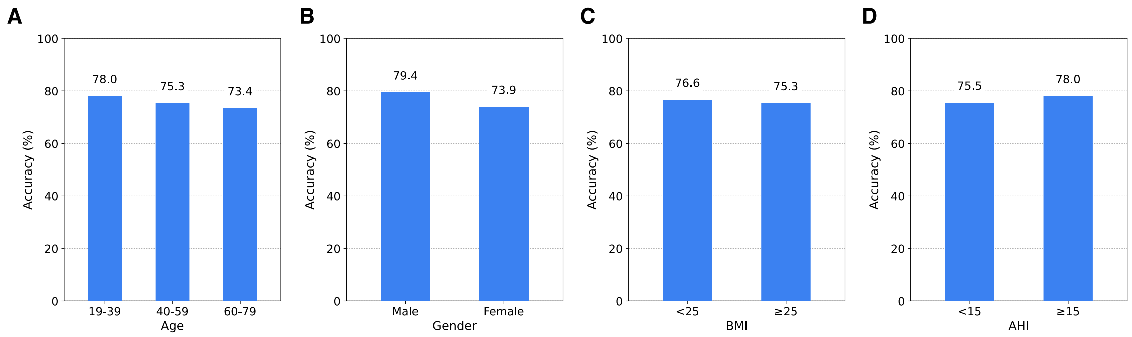

Figure S2. Accuracy of the HomeSleepNet model on different groups of demographic characteristics: (A) age, (B) gender, (C) BMI, (D) apnea-hypopnea index (AHI).
